# Multiple Roles of 25-Hydroxycholesterol in Lipid Metabolism, Antivirus Process, Inflammatory Response, and Cell Survival

**DOI:** 10.1155/2020/8893305

**Published:** 2020-11-19

**Authors:** Qin Cao, Zhongzhong Liu, Yan Xiong, Zibiao Zhong, Qifa Ye

**Affiliations:** ^1^Zhongnan Hospital of Wuhan University, Institute of Hepatobiliary Diseases of Wuhan University, Transplant Center of Wuhan University, Hubei Key Laboratory of Medical Technology on Transplantation, Engineering Research Center of Natural Polymer-based Medical Materials in Hubei Province, Wuhan, China 430071; ^2^The Third Xiangya Hospital of Central South University, Research Center of National Health Ministry on Transplantation Medicine Engineering and Technology, Changsha, China 410013

## Abstract

As an essential lipid, cholesterol is of great value in keeping cell homeostasis, being the precursor of bile acid and steroid hormones, and stabilizing membrane lipid rafts. As a kind of cholesterol metabolite produced by enzymatic or radical process, oxysterols have drawn much attention in the last decades. Among which, the role of 25-hydroxycholesterol (25-HC) in cholesterol and bile acid metabolism, antivirus process, and inflammatory response has been largely disclosed. This review is aimed at revealing these functions and underlying mechanisms of 25-HC.

## 1. Introduction

Cholesterol is a member of the sterol family that plays essential roles in a variety of biological processes [1]. Under physiological state, cholesterol is mainly metabolized into bile acids and steroid hormones such as estrogens and androgens. In addition, cholesterol is also the crucial component of membrane lipid rafts [2]. In the past years, the function of this basic and pleiotropic molecule has been deeply studied. It is then realized that the derivatives of this precursor are implicated in a broad of physiological processes, such as cholesterol metabolism, antivirus process, and inflammatory and immune response, and are involved in a series of diseases development, such as atherosclerosis, neurodegeneration disease, inflammatory bowel disease, and nonalcoholic liver disease [3–7]. Among these cholesterol metabolites, oxysterols are 27-carbon molecules that are formed via enzymatic or radical process adding an epoxide or ketone or an additional hydroxyl group in the sterol nucleus and/or a hydroxyl group in the side chain [8]. These compounds are much more chemically reactive than cholesterol and are involved in a wider range of physiological processes.

In the past decades, side-chain oxysterols including 24S-, 25-, and 27-HC have drawn much attention [9–12]. Both 24S-HC and 27-HC are responsible for excessive intracellular cholesterol efflux in extrahepatic tissues like brain and macrophages. When compared with cholesterol, 24S-HC and 27-HC have greater polarity, thus can be transported to liver for further metabolism [9]. 24S-HC and 27-HC are considered to be the major players in mediating cholesterol efflux from extrahepatic organs to liver [9]. Specifically, 24S-HC is merely produced in the brain, owing to the exclusively expression of cholesterol 24-hydroxylase (CYP46A1) [11].

When compared to 24S-HC and 27-HC, 25-HC is a minor side-chain oxysterol formed by cholesterol 25-hydroxylase (CH25H) [8]. As like other oxysterols, it was firstly thought that 25-HC had a potent ability to mediate cholesterol homeostasis. However, this hypothesis came into question when the cholesterol homeostasis was not affected on the condition of CH25H deficiency [13–15]. With the deep and broad investigations of this molecule, the veil of the involvement of 25-HC in antivirus process and inflammatory and immune response has been disclosed [12]. Over the past decades, the roles of 25-HC in cholesterol and bile acid metabolism, antivirus process, inflammatory and immune response, and survival signaling pathway have been widely investigated, and this review will depict the functions of 25-HC in these processes as comprehensive as possible.

## 2. 25-HC Production

**Figure 1:**
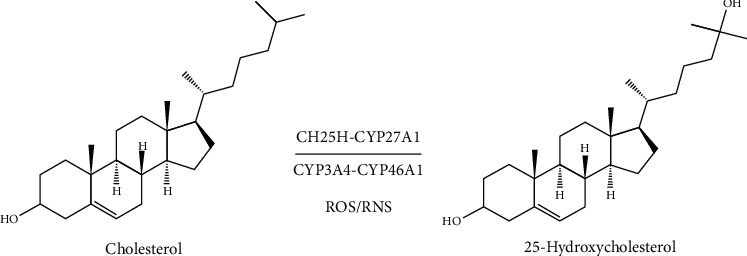
The production of 25-HC. Cholesterol can be catalyzed by enzymes CH25H, CYP3A4, CYP27A1, and CYP46A1 and reactive oxygen and nitrogen species (ROS/RNS) to 25-hydroxycholesterol.

25-HC is synthesized from cholesterol by the addition of a hydroxyl group at position 25-carbon. This reaction is catalyzed by CH25H, which is a member of a small family of enzymes that use oxygen and a di-iron cofactor to catalyze hydroxylation reaction [16]. CH25H is located in endoplasmic reticulum and is ubiquitously expressed in tissues, especially in macrophages [17]. There is recent study indicating that CH25H is highly expressed in mouse liver and peritoneal macrophages [18], although it was once considered that this protein was poorly expressed in healthy liver. In addition, a few of cytochromes (CYP3A4, CYP27A1, and CYP46A1) and even reactive oxygen and nitrogen species (ROS/RNS) can catalyze cholesterol to form this oxysterol [15, 19, 20] (Figure 1). However, the effect of these enzymes on 25-HC production in vivo is poorly investigated.

## 3. The Regulation of CH25H

**Figure 2:**
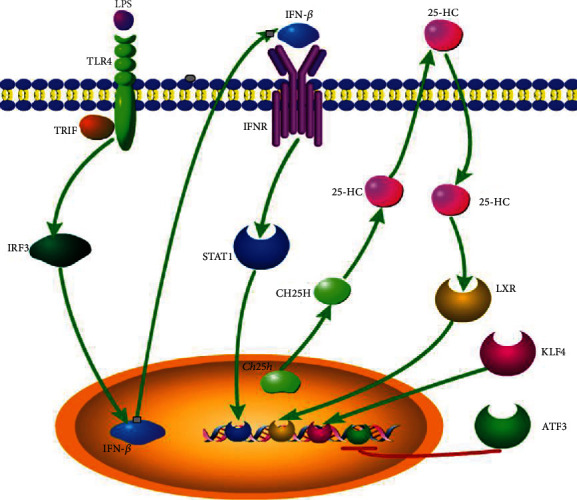
The regulation of CH25H expression. In LPS-stimulated macrophages, *Ch25h* is induced via a TLR4/IRF3/IFN-*β*/STAT1 signaling pathway. In addition, anti-inflammatory transcriptional factor KLF4 transactivates *Ch25h* in vascular endothelial cells. On the contrary, transcriptional factor ATF3 represses *Ch25h* transcription via directly binding to the *Ch25h* promotor. Furthermore, in hepatocytes and peritoneal macrophages, 25-HC induces CH25H expression in an LXR-dependent manner. Inflammatory cytokines IL-1*β*, TNF*α*, and IL-6 can also promote CH25H expression through the STAT1 transcription factor in virus-infected human macrophages (not shown).

Intracellular 25-HC content is mainly determined by CH25H activity; thus, regulation of CH25H is of great importance in 25-HC production. CH25H is a highly dynamically regulated enzyme and especially so in inflammatory conditions. Firstly, it was unexpectedly found that CH25H was strongly upregulated in lipopolysaccharide (LPS; endotoxin) stimulated macrophages in vitro, and this increased CH25H expression was independent of myeloid differentiation protein 88 (Myd88) signaling but dependent on toll-like receptor 4 (TLR4) signaling [21]. On the basis of this study, dendritic cells and macrophages were verified to be significant source of CH25H, and the TLR-mediated expression of CH25H was dependent on TIR-domain-containing adapter-inducing interferon-*β* (TRIF), production of type I interferons (IFNs), and signaling through the interferon production regulator (IFNR)/Janus kinase(JAK)/signal transducer and activator of transcription 1 (STAT1) pathway [17]. Subsequent study further validated the *Ch25h* as an INF-stimulated gene (ISG) via STAT1 pathway and 25-HC as the only macrophage synthesized and secreted oxysterol [22]. In addition, a cholesterol oxidation and efflux-related gene, krüppel-like factor 4 (KLF4) was recognized to be able to transactivate *Ch25h* in vascular endothelial cells and macrophages [23]. Recently, a study that is aimed at investigating the mechanisms regulating CH25H expression found that 25-HC itself was able to activate CH25H expression, thus forming a positive feedback loop, and this effect was dependent on liver X receptors (LXRs), which were receptors of 25-HC [18]. Furthermore, inflammatory cytokine interleukin-1*β* (IL-1*β*), tumor necrosis factor-*α* (TNF*α*), and IL-6 can also promote CH25H expression through the STAT1 transcription factor in virus-infected human macrophages [24]. On the contrary, activating transcription factor 3 (ATF3) was reported to be a negative regulator of *Ch25h* gene by directly binding to the promoter of *Ch25h* and epigenetically repressing *Ch25h* expression [25] (Figure 2).

## 4. The Receptors and Binding Proteins of 25-HC

The past years' investigations have shown that 25-HC is far more than just a kind of cholesterol metabolite. This molecule is an active mediator in a variety of physiological process. As an endogenous ligand, 25-HC binds to a strand of receptors [8, 26], including nuclear receptors LXRs [27, 28], retinoic acid receptor- (RAR-) related orphan receptors (ROR) [29, 30] and the estrogen receptor *α* (ER*α*) [31], and membrane receptor G protein-coupled receptor 183 (GPR183, also known as EBI2 for Epstein Barr virus-induced G protein-coupled receptor 2) [32, 33](Table 1). As the most broadly studied receptors, LXRs consist of two isoforms, LXR*α* (NR1H3) and LXR*β* (NR1H2). LXR*α* is expressed mainly in adipose tissue, liver, and intestine, with the highest in liver, while LXR*β* is ubiquitously expressed [34, 35]. 25-HC-activating LXRs are involved in a broad spectrum of physiological processes, such as cholesterol homeostasis and inflammatory response [35]. RORs are another family of nuclear receptor with three subtypes, ROR*α* (NR1F1), ROR*β* (NR1F2), and ROR*γ* (NR1F3), with ROR*γ* having two isoforms, ROR*γ*1 and ROR*γ*t [36, 37]. 25-HC has been described as an inverse agonist of ROR*α* and ROR*γ* [38, 39]. However, there is also a study indicating that 25-HC may have some agonistic activity to ROR*γ* [30]. 25-HC-activating ROR*γ*t is an essential transcription factor of T helper 17 (Th17) cell differentiation [30]. In addition, 25-HC was also shown to be an agonist of ER*α*-mediating gene expression changes and growth responses in breast and ovarian cancer cells [31]. Oxysterol-activating membrane receptor GPR183 directs immune cell migration [40], and 25-HC was demonstrated to be one of its agonists [32, 33]. In fact, the most potent GPR183 endogenous agonist is 7*α*,25-hydroxycholesterol (7*α*,25-HC), which is a 25-HC metabolite, catalyzed by oxysterol 7*α*-hydroxylase (CYP7B1) [32, 33].

In addition to receptors, 25-HC is able to bind some proteins binding oxysterols, including the insulin-induced gene protein (INSIG), Niemann-Pick protein (NPC), the oxysterol-binding protein family (OSBP related, OSBPL, or ORPs), and steroidogenic acute regulatory-related lipid transfer (START) domain proteins [8] (Table 1). INSIG is a regulatory protein for the sterol regulatory element-binding protein (SREBP), which regulates the expression of enzymes involved in cholesterol biosynthesis [41]. NPC1 is a membrane glycoprotein which resides primarily in the late endosomes and transiently in lysosomes [42], and 25-HC treatment was found to recover cholesterol clearance in lysosomal induced by NPC1 deficiency [43, 44]. OSBP and its related proteins are a family of lipid transfer proteins (LTPs), involving in lipid metabolism and signal transduction [45]. 25-HC-activating ORP8 suppresses ATP-binding cassette transporter (ABCA1), which mediates phospholipid and cholesterol efflux and inhibits macrophage cholesterol efflux [46]. In addition, ORP8 may be implicated in 25-HC-induced apoptosis of the hepatoma cell lines, HepG2 and Huh7, via the endoplasmic reticulum (ER) stress response pathway [47]. START domain is a protein module of approximately 210 residues that binds lipids, including sterols [48]. Fifteen mammalian proteins, STARD1-STARD15, possess a START domain. 25-HC can bind to STARD4 and STARD5 [49, 50], indicating their role in the maintenance of cellular cholesterol homeostasis.

## 5. Cholesterol and Bile Acid Metabolism

**Figure 3:**
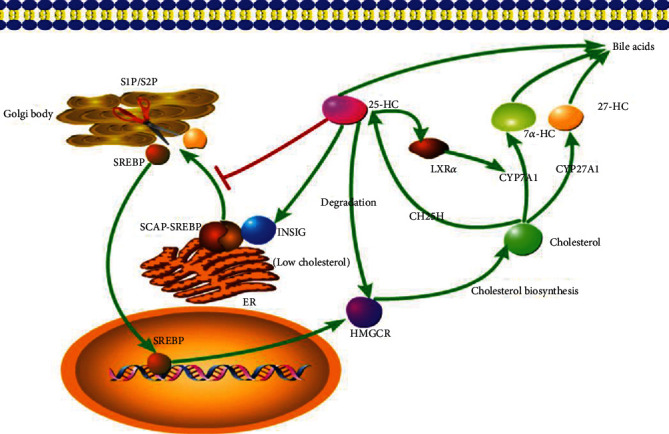
The regulation of 25-HC in cholesterol metabolism. Transcriptional factor SREBP controls the rate-limiting enzyme HMGCR. When the cholesterol level is low, the SREBP is escorted by SCAP from ER to Golgi body, in which this complex is cleaved by site-1 protease (S1P) and site-2 protease (S2P), and then the SREBP is released. 25-HC retains SREBP in ER via binds to an anchor protein INSIG in ER and suppresses SREBP translocation. Furthermore, 25-HC promotes HMGCR ubiquitylation and proteasomal degradation. 25-HC can also promote the CYP7A1 expression via activating transcriptional factor LXR*α*.

As a primary cholesterol metabolite, 25-HC mediates cholesterol biosynthesis, uptake, and efflux (Figure 3). The cholesterol homeostasis is controlled by a negative feedback loop by cholesterol itself and its derivatives, oxysterols, with the latter ones having more potent ability to suppress cholesterol biosynthesis [51, 52]. Transcriptional factor SREBP mediates the expression of cholesterol biosynthesis rate-limiting enzyme 3-hydroxy-3-methylglutaryl-CoA reductase (HMGCR) and many other related enzymes [53, 54]. SREBPs are retained in the ER in its inactive form. To become active, SREBPs must move from the ER to the Golgi by the multitransmembrane SREBP cleavage-activating protein (SCAP) [54]. The ER membrane protein, INSIG, with the interaction of SCAP keeps the SREBP-SCAP complex remaining in the ER [41]. 25-HC can bind to INSIG, thus keeping SREBPs inactive and inhibiting cholesterol synthesis [55]. In addition, 25-HC can directly reduce the level of the rate-limiting enzyme, HMGCR, by promoting its ubiquitylation and proteasomal degradation [56].

In addition to suppressing biosynthesis, 25-HC can also promote the cholesterol to be catalyzed to bile acids and intracellular cholesterol efflux. These effects are mainly dependent on LXRs [57]. 25-HC-activating LXR*α* in hepatocytes induces the rate-limiting enzyme in the classic bile acid synthetic pathway, cholesterol 7*α*-hydroxylase (CYP7A1) [57], promoting cholesterol to be converted to bile acids. In macrophages, LXRs can induce the expression of ATP-binding cassette subfamily members A1 (ABCA1) and G1 (ABCG1), which are responsible for reverse cholesterol transport, eliminating excessive intracellular cholesterol [57, 58].

Above all, it seems clear that 25-HC suppresses the cholesterol biosynthesis and promotes the intracellular cholesterol efflux through a variety of mechanisms. However, in vivo study using *Ch25h* knockout mice showed that CH25H and 25-HC deficiency did not affect whole cholesterol metabolism, which questioned the mediation of 25-HC in cholesterol homeostasis [13, 14]. These inconsistent conclusions led to the hypothesis that 25-HC might play a role in cholesterol catabolism in a districted area and limited cells, not affecting the whole cholesterol homeostasis [12].

Bile acids are exclusively synthesized in liver through two distinct routes. In addition to the classical one initiated by CYP7A1, the so-called alternative pathway started with cholesterol 27-hydroxylase (CYP27A1) followed by oxysterols 7*α*-hydroxylase (CYP7B1) [13, 59]. 25-HC is also a precursor of bile acids, although it is not so impressive as 27-hydroxycholesterol (27-HC). The bile acids derived from 25-HC count less than 5% in total per day [60]. And as like cholesterol metabolism, CH25H and 25-HC deficiency does not affect the whole bile acids homeostasis [13].

## 6. Antivirus Effects

Oxysterols link the bridge between lipid metabolism and innate and adaptive immune response [61]. As an ISG, *Ch25h* is highly induced in virus infection, and 25-HC is impressive for its potent ability to inhibit virus invasion through a strand of mechanisms [62].

25-HC has a broad antivirus spectrum, including enveloped viruses and nonenveloped viruses [22, 63–72]. The enveloped viruses mainly consist of murine cytomegalovirus (MCMV), vesicular stomatitis virus (VSV), West Nile virus (WNV), the human immunodeficiency viruses (HIV), influenza virus, murid herpesvirus 68 (MHV68) and Ebola virus, Rift Valley fever virus (RVFV), Russian spring-summer encephalitis virus (RSSEV), Nipah virus, herpes simplex virus 1 (HSV-1), varicella-zoster virus (VZV), hepatitis B virus (HBV), hepatitis C virus (HCV), and the recently epidemic coronavirus disease 2019 (COVID-19) [22, 63–68]. The nonenveloped viruses include poliovirus, the encephalomyocarditis virus (EMCV), human papillomavirus type 16 (HPV-16), human rotavirus (HRoV), and human rhinovirus (HRhV) [69–72].

25-HC exhibits its antivirus function via a variety of mechanisms. Cholesterol metabolism is of great significance for viruses invading into the cells, the adsorption, entry, assembly, budding, and release of some viruses preferentially occurring in cholesterol-enriched microdomains (“lipid rafts”) of the cell membrane, especially the enveloped viruses [73]. 25-HC can directly change the position, orientation, and solvent accessibility of cholesterol in membrane, thus blocking the virus entry. Furthermore, 25-HC may insert into the cell membrane, changing the stability and integrity of cholesterol-enriched cytomembranes, inhibiting the fusion of virus and host cell membrane [74]. In addition to mediate the cell membrane status, 25-HC can also directly inhibit the virus replication. For example, the nonstructural protein 1 alpha (nsp1*α*) is an essential protein for porcine reproductive and respiratory syndrome virus (PRRSV) replication. CH25H/CH25H-M could degrade nsp1*α* through the ubiquitin-proteasome pathway [75]. Furthermore, high micromolar amounts of 25-HC-induced integrated stress response in host cells were also demonstrated to suppress the virus replication [63]. Lastly, *Ch25h* and 25-HC are crucial mediators in innate and adaptive immune response, and 25-HC treatment-induced inflammatory factors' release and the mediation in immune response are reported to inhibit virus invasion as well [22, 76].

## 7. Inflammatory Response

**Figure 4:**
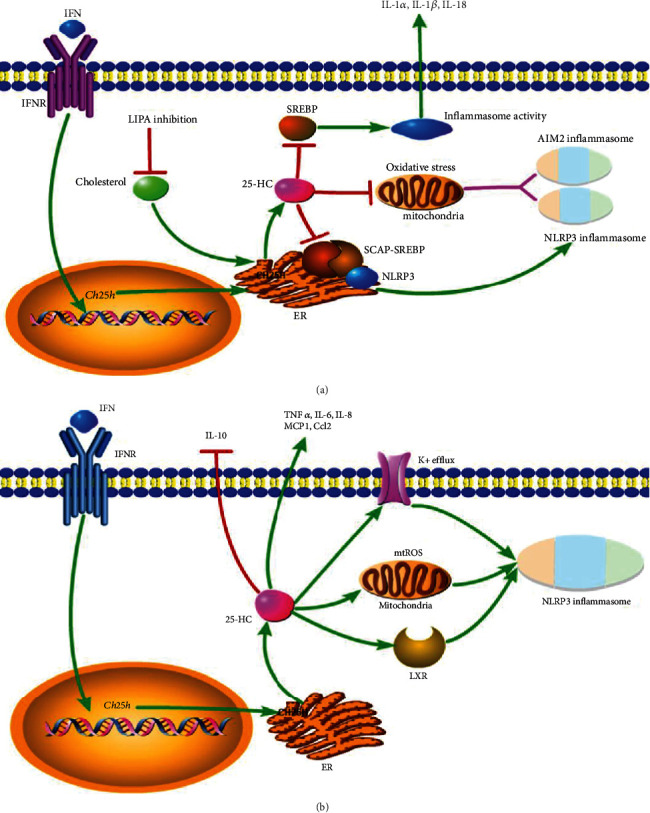
(a) The anti-inflammatory effect of 25-HC. 25-HC reduces IL-1 family (IL-1*α*, IL-1*β*, and IL-18) release and inflammasome activity via inhibiting SREBP. 25-HC decreases NLRP3 and AIM2 inflammasome formation by reducing mitochondria oxidative stress. Furthermore, NLRP3 associates with SCAP and SREBP2 to form a ternary complex which translocated to the Golgi apparatus adjacent to a mitochondrial cluster for optimal inflammasome assembly, and 25-HC inhibits this process. (b) Proinflammatory effect of 25-HC. 25-HC amplifies the expression of proinflammatory factors (TNF*α*, IL-6, IL-8, MCP1, and Ccl2) and reduces the anti-inflammatory factor IL-10. 25-HC promotes robust NLRP3 inflammasome assembly and activation via potassium efflux, mitochondrial ROS, and LXR-mediated pathways in brain.

Does 25-HC amplify inflammatory response? It is a question. On the one hand, 25-HC is able to suppress interleukin-1 (IL-1) family cytokine production, such as IL-1*α*, IL-1*β*, and IL-18 [77, 78]. Via using *Ch25h* knockout mice, it was reported that 25-HC acted by antagonizing SREBP processing to reduce IL-1*β* transcription and to broadly repress IL-1-activating inflammasomes [79]. However, the specific mechanism by which the SREBPs promote IL-1*β* transcription is not clear. The authors speculated that it might be induced by cellular lipid content alteration caused by altered SREBP activity [12]. Another study verified the effect of cellular lipid content on NLR family pyrin domain containing 3 (NLRP3) inflammasome activation. This study showed that reduced synthesis of 25-HC resulting from the lysosomal acid lipase (LIPA) inhibition, which hydrolyzes cholesteryl esters to free cholesterol for 25-HC synthesis in macrophages, contributed to defective mitochondria-associated membrane (MAM) leading to mitochondrial oxidative stress-induced NLRP3 inflammasome activation [80]. In addition, SCAP escorts both NLRP3 and SREBP2 by forming a ternary complex, and 25-HC inhibited NLRP3 inflammasome formation via maintaining SCAP in ER [81]. Furthermore, 25-HC was found to suppress another common inflammasome activation. It was reported that high cholesterol content in macrophages was enough to activate the DNA sensor protein absent in melanoma 2 (AIM2) inflammasome by inducing impaired mitochondrial metabolism and mtDNA release, and 25-HC was able to maintain mitochondrial integrity and prevent AIM2 inflammasome activation in activated macrophages, in which the CH25H was upregulated [82] (Figure 4(a)). However, there is a study showing that 25-HC promotes the caspase-1-dependent cell death of colon cancer cells via activating LXR*β*, but not LXR*α* [83]. And subsequent study found that 25-HC promoted robust NLRP3 inflammasome assembly and activation via potassium efflux, mitochondrial reactive oxygen species (ROS), and LXR-mediated pathways in X-linked adrenoleukodystrophy (X-ALD) [84] (Figure 4(b)). These controversial conclusions are hard to explain. It might be speculated that the function of 25-HC in NLRP3 activation may follow a tissue and cell-dependent manner.

Except for activating inflammasome, 25-HC promotes proinflammatory cytokines and chemokines, such as tumor necrosis factor-*α* (TNF*α*), interleukin-6 (IL-6), interleukin-8 (IL-8), monocyte chemoattractant protein-1 (MCP1), and C-C motif chemokine ligand 2 (Ccl2) [85–90]. In addition, it is also able to suppress the secretion and production of anti-inflammatory factor interleukin-10 (IL-10). In human CD4 T cells, 25-HC reduces IL-10 production via decreasing the master transcriptional regulator of IL-10, c-Maf. In IL-27-induced type 1 regulatory T (TR1) cells, 25-HC acts as a negative regulator of TR1 cells in particular of IL-10 secretion via LXR signaling [91, 92].

Two transcriptional factors, nuclear factor kappa-light-chain-enhancer of activated B cells (NF-*κ*B) and Activator protein 1 (AP-1) are downstream effectors while 25-HC exerting its proinflammatory function [88, 93, 94]. And a variety of downstream signaling pathways are involved in this process. In mouse peritoneal macrophages and human umbilical cord vein endothelial cells, 25-HC induces retinoic inducible gene I (RIG-I). RIG-I transduces the signal to downstream molecules, mitochondrial antiviral-signaling protein (MAVS), transforming growth factor-*β*-activated kinase 1 (TAK-1), and mitogen-activated protein kinase (MAPK/ERK/P38/JNK), leading to the activation of NF-*κ*B and AP-1, inducing IL-8 production [95]. Furthermore, 25-OH triggers the activation/phosphorylation of the AP-1 component c-Jun and, consistently, increases the transcriptional activity of AP-1 [96].

## 8. 25-HC and Cell Survival

### 8.1. Autophagy

Autophagy is an evolutionarily ancient process whereby eukaryotic cells eliminate disposable or potentially dangerous cytoplasmic material to support bioenergetic metabolism and adapt to stress. It remains controversial of the function of 25-HC in mediating autophagy which is dependent on cell types. Lysosomal cholesterol accumulation sensitizes hepatocytes to acetaminophen toxicity by impairing mitophagy, and 25-HC recovers hepatocyte mitophagy by decreasing lysosomal cholesterol accumulation [97]. However, in human glioblastoma cell line (U87-MG), 25-HC is ineffective to restore autophagy flux and to decrease apoptosis levels [98]. In non-small-cell lung cancer cells (H1299), 25-HC is reported to induce cell death via attenuating autophagy [99].

### 8.2. Apoptosis

25-HC is reported to induce cell apoptosis in dose-dependent manner; however, the underlying mechanisms are poorly understood. Endoplasmic reticulum (ER) stress resulting from 25-HC seems to play a key role in this oxysterol-mediated proapoptotic effect. In macrophages, oxysterol-binding protein-related protein 4L (ORP4L) coexpresses and forms a complex with G*α*q/11 and phospholipase C- (PLC-) *β*3. ORP4L facilitates PLC*β*3 activation, IP3 production, and Ca2+ release from the endoplasmic reticulum. Through this mechanism, ORP4L sustains antiapoptotic Bcl-XL expression through Ca2+-mediated c-AMP responsive element-binding protein transcriptional regulation and thus protects macrophages from apoptosis. However, excessive 25-HC disassembles these ORP4L/G*α*q/11/PLC*β*3 complexes, reducing PLC*β*3 activity, IP3 production, and Ca2+ release, resulting in macrophage apoptosis [100, 101]. In hepatic cell HepG2 and Huh7, 25-HC facilitates apoptosis via enhancing endoplasmic reticulum (ER) stress, and oxysterol-binding protein-related protein 8 (ORP8) is involved in 25-HC-mediated ER stress and hepatic cell apoptosis [47]. ORP8 knockdown rescues this effect. Neutral cholesterol ester hydrolase 1 (Nceh1) is a hydrolysis enzyme that dissolves 25-HC ester to free 25-HC. Incubating Nceh1-deficient thioglycollate-elicited peritoneal macrophages (TGEMs) with 25-HC caused massive accumulation of 25-HC ester in the endoplasmic reticulum (ER) due to its defective hydrolysis, thereby activating ER stress signaling and subsequent apoptosis [102]. In addition, 25-HC is able to induce apoptosis of vascular smooth muscle cells (VSMC) by controlling mitochondrial Bax translocation and ROS formation in a soluble adenylyl cyclase (sAC)/protein kinase A- (PKA-) dependent pathway [103].

## 9. Conclusions and Perspective

This review depicts the function of a kind of oxysterol, 25-HC, in cholesterol and bile acid metabolism, antivirus process, inflammatory response, and cell survival, especially in autophagy and apoptosis. It is astonishing that such a small molecule is involved in so broad variety of physiological processes. The only difference between 25-HC and another two common primary oxysterols, 27-HC and 24S-HC, lies in the carbon position, to which a hydroxyl group is added. However, 25-HC has much more potent antivirus ability than the other two. The reason is not clear yet. Owing to the broad antivirus spectrum and potent antivirus effect, there is a high possibility that 25-HC will be used as a drug in antivirus treatment. And studies exploring the specific mechanisms of 25-HC antivirus effect can be anticipated. Furthermore, the dual effects of 25-HC in proinflammatory and anti-inflammatory lead to the hypothesis that 25-HC may not be a simple positive or negative regulator in inflammatory response, but a mediator keeping inflammatory response in an accepted degree. In addition, the final phenotype in inflammatory response may be 25-HC amount and tissue dependent. Thus, more in vivo studies are needed to tell us the whole story.

## Figures and Tables

**Table 1:** The receptors and binding proteins of 25-HC.

Receptors	Role	Functions
Liver X receptors (LXR*α*, LXR*β*)	LXR*α* agonist LXR*β* agonist	1. Negatively regulates cholesterol biosynthesis and promotes cholesterol efflux [27, 28, 34, 35] 2. Inflammatory regulation [35] 3. Promotes pyroptosis [83]
Retinoic-related orphan receptors (ROR*α*, ROR*β*, ROR*γ*)	ROR*α* ligand ROR*γ* agonist	Th17 cell differentiation [30, 36–39]
Estrogen receptor *α* (ER*α*)	Agonist	Mediates gene expression changes and growth responses in breast and ovarian cancer cells [31]
G protein-coupled receptor 183 (GPR183, EBI2)	Agonist	Directs immune cell migration [32, 33]
Proteins binding oxysterols		
Insulin-induced gene protein (INSIG)	Ligand	Maintains SREBP in the ER and inhibits cholesterol biosynthesis [41]
Niemann-Pick protein C1 (NPC1)	Ligand	Cholesterol clearance in lysosomal [42–44]
Oxysterol-binding protein family 8 (ORP8)	Ligand	1. Cholesterol efflux in macrophages [46] 2. Induces apoptosis of the hepatoma cell lines [47]
Steroidogenic acute regulatory-related lipid transfer (START) domain proteins	Ligand	Maintenance of cellular cholesterol homeostasis [48–50]
